# Comparative effectiveness trial comparing MyPlate to calorie counting for mostly low-income Latino primary care patients of a federally qualified community health center: study design, baseline characteristics

**DOI:** 10.1186/s12889-019-7294-z

**Published:** 2019-07-24

**Authors:** Lillian Gelberg, Melvin W. Rico, Dena R. Herman, Thomas R. Belin, Maria Chandler, Evangelina Ramirez, Stephanie Love, William J. McCarthy

**Affiliations:** 1 Department of Family Medicine, David Geffen School of Medicine at UCLA, University of California, 10880 Wilshire Blvd, Ste 1800, Los Angeles, CA 90024 USA; 2 Charles R. Drew/UCLA Medical Education Program, Los Angeles, USA; 3 UCLA Fielding School of Public Health, 51-267 CHS, mc 177220, 650 Charles Young Drive, Los Angeles, CA 90095 USA; 4 The Children’s Clinic of Long Beach Patient Representative, 1715 E. Anaheim St., Apt. 2, Long Beach, CA 90813 USA; 5 Family and Consumer Sciences, College of Health and Human Development, SQ 200M 18111 Nordhoff Street, Northridge, CA 91330 USA; 6 TCC, 701 East 28th Street, Suite 200, Long Beach, CA 90806 USA; 7 TCC, 19327 Broadacres Ave, Carson, CA 90746 USA; 8 UCLA Fielding School of Public Health, A2-125 CHS, mc 690015, 650 Charles Young Drive, Los Angeles, CA 90095 USA

**Keywords:** Latino, Primary care, Obesity treatment, Satiety, Satiation, Nativity, Behavior change, Community health workers

## Abstract

**Background:**

Primary care-based behavior change obesity treatment has long featured the Calorie restriction (CC), portion control approach. By contrast, the MyPlate-based obesity treatment approach encourages eating more high-satiety/high-satiation foods and requires no calorie-counting. This report describes study methods of a comparative effectiveness trial of CC versus MyPlate. It also describes baseline findings involving demographic characteristics and their associations with primary outcome measures and covariates, including satiety/satiation, dietary quality and acculturation.

**Methods:**

A comparative effectiveness trial was designed to compare the CC approach (*n* = 130) versus a MyPlate-based approach (*n* = 131) to treating patient overweight. Intervenors were trained community health workers. The 11 intervention sessions included two in-home health education sessions, two group education sessions, and seven telephone coaching sessions. Questionnaire and anthropometric assessments occurred at baseline, 6- and 12 months; food frequency questionnaires were administered at baseline and 12 months. Participants were overweight adult primary care patients of a federally qualified health center in Long Beach, California. Two measures of satiety/satiation and one measure of post-meal hunger comprised the primary outcome measures. Secondary outcomes included weight, waist circumference, blood pressure, dietary quality, sugary beverage intake, water intake, fruit and vegetable fiber intake, mental health and health-related quality of life. Covariates included age, gender, nativity status (U.S.-born, not U.S.-born), race/ethnicity, education, and acculturation.

**Analysis:**

Baseline characteristics were compared using chi square tests. Associations between covariates and outcome measures were evaluated using multiple regression and logistic regression.

**Results:**

Two thousand eighty-six adult patients were screened, yielding 261 enrollees who were 86% Latino, 8% African American, 4% White and 2% Other. Women predominated (95%). Mean age was 42 years. Most (82%) were foreign-born; 74% chose the Spanish language option. Mean BMI was 33.3 kg/m^2^; mean weight was 82 kg; mean waist circumference was 102 cm. Mean blood pressure was 122/77 mm. Study arms on key baseline measures did not differ except on dietary quality and sugary beverage intake. Nativity status was significantly associated with dietary quality.

**Conclusions:**

The two treatment arms were well-balanced demographically at baseline. Nativity status is inversely related to dietary quality.

**Trial registration:**

NCT02514889, posted on 8/4/2015.

## Background

In the United States, 31.8% of adults are overweight but not obese (between 25 and 29.9 kg/m^2^) and an additional 39.8% are obese (BMI > 30 kg/m^2^) [1, 2]. Non-Hispanic Black women appear to be at especially high risk (56.1% obese), followed closely by Hispanic women (48.4% obese) [3]. The lifetime medical cost burden of overweight and obesity is substantial and could be reduced through early treatment and prevention [4]. Obesity increases the risk of cardiovascular disease (CVD) via a variety of mechanisms [5, 6]. The American Heart Association and other organizations recommend weight loss and regular physical activity for the prevention and treatment of obesity-related diseases [7–10]. More particularly, abdominal obesity increases the risk of type 2 diabetes, especially in ethnic minority groups [11, 12]. Hispanics and African Americans are particularly at risk of having type 2 diabetes [13]. Lifestyle change efforts promoting weight loss in patients with obesity through increased physical activity and healthier food choices can reduce risk of type 2 diabetes [14, 15].

Two rigorous trials of successful behavioral change weight loss interventions administered to overweight, low-income patients recruited from community health centers were reported in 2011 [16, 17]. Both trials featured a lifestyle change intervention with no adjuncts such as meal replacement products or use of weight loss drugs. One of these lifestyle interventions featured a conventional energy restriction approach to weight loss but also featured the Dietary Approach to Stop Hypertension (DASH) diet [18–20], a model dietary pattern explicitly recommended by the *Dietary Guidelines for Americans* for consumption by all healthy Americans, regardless of weight status [21]. The other lifestyle intervention was patterned after the energy restrictive, behavioral intervention approach used in the Diabetes Prevention Program (DPP) [15]. The DPP lifestyle change approach seeks to create a calorie deficit in overweight patients by increasing energy expenditure in daily physical activity and limiting daily intake of calories. In the DPP, this approach yielded 7% weight loss over 2.8 years and a 58% reduction in risk of diabetes compared to usual care [15]. In the 2011 trials, however, the DASH-like diet yielded a 5.4 (95% CI, 4.0, 6.8) kg weight loss at 1 year compared to the 3.4 (95% CI, 2.2, 4.6) kg weight loss observed in the DPP-like intervention. This difference in impact of the two weight loss approaches resembled the results of another trial where a fruit and vegetable-supplemented fat-restricted diet yielded slightly better 1-year weight loss than a standard fat-restrictive weight loss regimen [22]. The commercial weight loss program, Weight Watchers, has achieved success in part by encouraging clients to eat more fruits and vegetables in addition to restricting total daily calorie intake [23, 24]. Other research is confirming the weight control-facilitating benefits of daily consumption of fresh fruit and vegetables [25, 26].

**Fig. 1:**
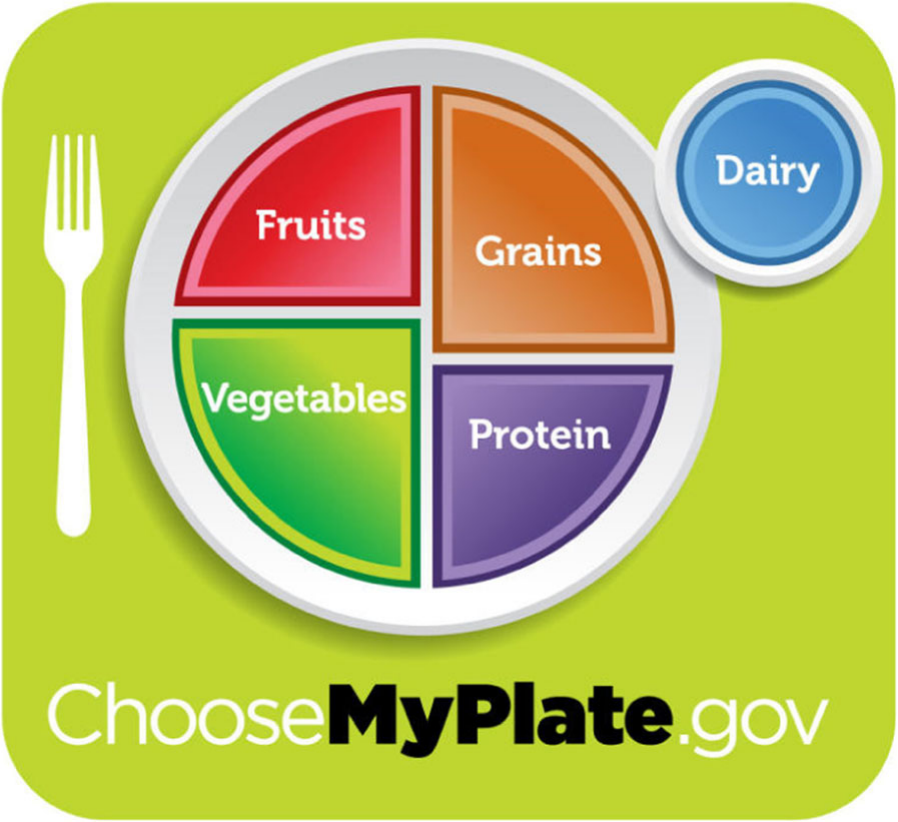
U.S. Department of Agriculture MyPlate icon, as downloaded from www.choosemyplate.gov

Both the DPP and the DASH lifestyle change approaches were designed to reduce daily energy intake. The classical calorie counting (CC) approach (See Table 1 for a detailed comparison of features) focuses on using psychological self-regulatory strategies to motivate adherence, including social support, self-reward to maintaining desirable weight, and encouragement by trusted counselors but makes little attempt to alter participant food choices in order to minimize post-meal hunger [27]. Consistent predictors of weight loss maintenance under the CC approach are dietary restraint and disinhibition, neither of which are thought to be dependent on the nature of one’s food choices but rather are thought to be largely a function of participant motivation [28]. By contrast the DASH-diet investigators [17] focused their lifestyle change efforts on getting patients to make major changes in daily food choices [29]. A defining feature of the DASH dietary pattern (see Table 1 for details) is that it encourages daily intake of twice the quantity of fruits and vegetables as is typically consumed in the usual American diet [30]. Despite the priority that the DASH-diet investigators placed on weight loss [17], participants were encouraged to increase their intake of minimally processed fruits and vegetables. The recommendation to eat daily a greater quantity of minimally processed fruits and vegetables has recently been given more prominence as one of seven dietary recommendations associated with www.ChooseMyPlate.gov [31], the federal initiative that replaced the food pyramid with a food plate as the nation’s leading nutrition education icon (see Fig. 1). MyPyramid, the predecessor to MyPlate, included 6 food groups versus 5 for MyPlate, required knowing what a standard serving size was for each food group, encouraged consumption of more grain-rich foods than fresh produce and seemed to encourage consumption of refined oils, sweets and other problem food components by including them at the top of the pyramid. MyPlate is simpler, focusing on only four food groups on the plate and dairy on the side, showing fruits and vegetables as occupying twice the space on the plate as (whole) grains, and highlighting that only one quarter of the plate should be occupied by high-quality protein sources, including legumes and nuts. The specific recommendation is for Americans to fill half their plate with minimally processed fruits and vegetables (fruit juice not included). Counter-intuitively, interventions that induce overweight individuals to increase their consumption of minimally processed fruits and vegetables are consistently (but not always) associated with reduced body weight at 6-months [18], 12-months [22], 2-years follow-up [32] and 4-years follow-up [33]. The exceptions are fruit and vegetable-based interventions that include 100% fruit juices [34]. Increased obesity risk has been associated with consuming fruit in the form of fruit juice [35]. Fruit and vegetable juices typically exclude the dietary fiber that had been in the original fruit/vegetable [35], which thereby removes substrate that could have fueled commensal gut microbial generation of short chain fatty acids [36]. Increased short chain fatty acids, in turn, stimulate increased satiety signaling, thereby reducing appetite [37]. An additional satiating benefit of consuming more fruits and vegetables, minimally processed, is the lower energy density of minimally processed fruits and vegetables (they are 70–94% by weight water), permitting DASH trial participants to increase their total daily gram-weight intake of food by 24% even while decreasing their daily energy intake by 10% [38].

**Table 1:** Defining features of the calorie counting and MyPlate approaches to desirable weight loss

Feature	Historical approaches		Experimental approaches	
	Diabetes Prevention Program^a^	Dietary Approach to Stop Hypertension (DASH)^b^	Calorie Counting approach (CC)	MyPlate approach (MyP)
Restricts total calories/day	Yes	No	Yes	No
Requires monitoring of calorie intake throughout the day	Yes	No	Yes	No
Recommends 8+ servings of fruits and vegetables/day	No	Yes	No^c^	Yes
Recommends limits on sodium intake	No	Yes	No	Yes
Recommends limits on saturated fat intake	Yes	Yes	Yes	Yes
Recommends limits on sugary beverage consumption	No	Yes	Yes^d^	Yes
Recommends limiting snacks and sweets even if within calorie limits	No	Yes	No	Yes
Requires restraint when still hungry after eating full meal	Yes	No	Yes	No
Recommends accompanying exercise ~ 30+ min. MVPA*/ day	Yes	Yes	Yes	Yes

* MVPA = Moderate to vigorous (aerobic) physical activity

^a^Knowler WC, Barrett-Connor E, Fowler SE, et al. Reduction in the incidence of type 2 diabetes with lifestyle intervention or metformin. *N Engl J Med.* 2002;346 (6):393–403

^b^Appel LJ, Moore TJ, Obarzanek E, et al. A clinical trial of the effects of dietary patterns on blood pressure. *N Engl J Med.* 1997;336 (16):1117–1124

^c^The CC approach encouraged eating more foods low in energy density, especially fruits and vegetables, but the encouragement did not include a target of 8 servings/day

^d^The CC approach encouraged limits on weekly consumption of sugary beverages at the behest of community dietitians who otherwise followed a conventional DPP-like CC approach

While both the DPP and DASH dietary approaches reduced excess body weight short term, the ability of diverse patients to maintain these approaches long-term remains to be determined. Short-term emotional well-being is typically increased during adherence to calorie restriction regimens [39–41] but is usually not enough to sustain the desired weight loss beyond 5 years [42]. People who successfully lost 30 kg or more of excess weight without use of adjuncts (e.g., meal replacements, anorexigenic drugs) and maintained this loss for five or more years report that the effort required to keep this weight off diminishes with time [43], but the mechanism that explains why reduced effort is needed to maintain desired weight loss with time is unknown. This study was partly designed to address this gap by focusing on the satiety/hunger consequences of food choices and the downstream impact on quality of life and mental health. Previous research showed that a fruit and vegetable-supplemented weight loss program yielded less post-meal hunger in addition to greater weight loss at 1-year follow-up than a traditional fat- restrictive approach [22]. Previous research also showed that good adherence to a Mediterranean dietary pattern was associated with higher quality of life [44].

The investigators took several steps to adapt the DPP and DASH interventions to ensure that the intervention effects for either intervention condition could be sustained over the long term. One step was to replace the masters-level health educators with community health workers. The social modeling of Social Cognitive Theory [45, 46] and researchers’ experience [47] suggest that the predominantly low-income Latino immigrant patients comprising most of the study population can relate to Latino community health workers better than they can to bilingual but non-Latino masters or doctoral-level counselors [47]. African American type 2 diabetes patients as well as Latino patients have benefited from use of community health workers as behavior change agents [48, 49]. The Weight Watchers commercial weight loss program has long featured peer leaders as their weight loss counselors, with demonstrated success [50].

A second step was to fix the maximum number of coaching sessions at 11 contacts (2 in-home, 2 group education sessions, 7 telephone coaching sessions), to approach the number of individual-level contacts used in previous clinic-originated weight loss efforts [16, 17, 51–55]. This was done to expand opportunities for participant-coach problem-solving and participant trialing of specific lifestyle change strategies, and to capitalize on the motivational benefits of monitoring by health care professionals [56]. This level of contact is admittedly less than in the clinic-based study employing the DASH approach, which included 9 individual sessions, 3 phone contacts and 12 group sessions in the first 6 months [17]. The clinic-based study employing the DPP approach included 8 individual or phone contact sessions in the first 6 months [16].

A third step was to devote more intervention resources to ensuring that the participant’s home environment was optimally supportive of healthier lifestyle choices. Two thirds of calories are typically consumed in the home [57]. Both physical (e.g., type of food available) and social (e.g., support from family) factors in this setting have been associated with weight, dietary habits, and activity patterns [58, 59]. A fourth feature (in the MyPlate condition only) was the inclusion of taste-testing to induce participants to increase their liking for a greater range of palatable fruits and vegetables, minimally processed [33, 60–63]. A fifth feature was to conjoin the nutrition messages from both dietary approaches with the recommendations from the *Physical Activity Guidelines for Americans-2nd edition* [64]. For the MyPlate approach, increased daily physical activity was seen as a way to engender greater appetite for water-rich plant foods, minimally processed [65] and as a way to minimize intestinal inflammation [66], a moderator of fiber-induced satiety-signaling [67]. For the Calorie Counting (CC) approach, increased physical activity was seen as a way to increase the energy expenditure side of the energy balance equation [68].

The aims of this paper are to describe the design of a comparative effectiveness trial, comparing the CC versus MyPlate (MyP) approaches. This description includes the effect of community input on the design of the intervention, the overall study methods, the baseline findings regarding demographic characteristics, primary outcome measures and covariates, including satiety/satiation, nativity status, and acculturation.

## Methods

This study was designed to compare the intervention impact of two government-sanctioned weight control approaches on self-reported satiation/satiety, a patient-centered outcome, and on objectively assessed waist circumference, a conventional medical outcome. The MyPlate high-satiety/high-satiation approach to desirable weight loss was expected to yield increasing satiety over time, over and above whatever increases in satiety might be observed in the more traditional CC condition. The increased satiety, in turn, was expected to engender increased mental health and increased health-related quality of life, two correlates of long-term adherence to desirable lifestyle change [69, 70]. Even though the MyPlate arm was expected to yield greater satiety and less post-meal hunger then the CC arm, its initial rate of weight loss was expected to be slower but better sustained than the weight loss obtained through calorie restriction. Hence, the MyPlate arm was expected to be as effective in reducing body fat composition at 12 months follow-up as the more traditional calorie-restrictive CC weight loss approach because of disproportionate weight regain in the CC arm offsetting the CC arm’s expected superior weight loss in the short run [71].

### Community advisory board

Members of the Community Advisory Board (CAB) were identified and recruited with input from the medical director of the federally qualified health center (FQHC) hosting this trial, the study patient representatives, and the FQHC’s director of community outreach. In addition to the medical director and the patient representatives, the CAB included 15 members representing a broad cross-section of the Long Beach, California community, including physicians, dietitians, community gardening specialists, two patient representatives, a representative of the local YMCA, a pastor, a health educator, and ex officio, and the two UCLA principal investigators. The CAB first met in June 2014 to discuss the study aims, draft study protocol, and proposed assessment instruments.

### Focus groups and key stakeholder interviews

Focus groups (2 focus groups, 4 members per group) and 6 key stakeholder interviews were conducted to vet the community acceptability of the proposed intervention design and assessment instruments. Most of the focus group participants and key stakeholder interviewees were originally identified by members of the CAB. The investigators conducted two focus groups, one consisting of Spanish-speaking FQHC patients with obesity, the second consisting of English-speaking FQHC patients with obesity. The criteria for focus group participant selection were similar to the criteria expected to be used in recruiting study participants, to ensure comparability of demographic characteristics between focus group participants and study participants. The investigators also interviewed six community stakeholders, none of whom was an FQHC patient but all of whom were actively engaged members of the Long Beach community. The key stakeholder interviewees included a pastor, a community activist and other community leaders knowledgeable about the dietary practices and physical activity habits of Long Beach residents and knowledgeable about the health promotion resources available in Long Beach.

The discussion guide used to facilitate the focus group discussion and key stakeholder interviews asked six questions about the interlocutor’s attitudes about daily food choices, and five questions about their attitudes about physical activity. The discussion guide also included eight questions about strategies to improve both the quality of one’s daily food choices and weekly level of physical activity as well as outcomes that would matter to them in a weight reduction intervention designed for patients like them. The discussion guide preamble stated that the participants’ / interviewee’s answers would help the investigators design a better weight loss intervention for TCC patients who were overweight or obese and who wanted to lose some of their excess weight. The questions for the discussion guide were based on the investigators’ previous experience with promotora-led, home-based dietary interventions with Latinos. The focus group discussion lasted 1 h and took place in a community setting operated by the Long Beach Health Department. The key stakeholder interviews generally lasted 35 min. All participants were paid $20 cash following their participation. Six of the eight focus group participants were women; four of the six key stakeholder interviewees were women.

The facilitator of the focus groups was a Spanish-speaking bilingual, bicultural Latina graduate student with experience in the collection of qualitative data. The note taker was also a bilingual female graduate student. Although the discussions were audio-recorded, they were not transcribed. The content analyses used the notetaker’s notes as the source of the data. When questions arose concerning the meaning of the notetaker’s notes, the content analysts consulted the audio record. The analysis protocol used to sort the themes was based on the manual protocol described in Krueger & Casey [72].

### Pilot study

A 10-person pilot intervention study was carried out over 3 months featuring all of the planned intervention sessions (12 sessions initially). Two of the four community health workers hired for the full trial participated as change agents in the pilot study. Results of the pilot study confirmed the acceptability of all intervention features but the decision was made to reduce by one the number of telephone coaching sessions, from 8 to 7, because of the community health workers’ perception that there were diminishing returns as the number of phone calls increased. The home visits and group education sessions were well-received, however, and were therefore kept as originally designed.

### Comparative effectiveness trial

#### Study design

The investigators carried out a parallel group, randomized, controlled comparative effectiveness trial comparing MyPlate to Calorie Counting (CC). More specifically, the trial sought to resolve whether individuals should engage in portion control and restrict calories from all foods (as originally recommended by the Diabetes Prevention Program [15]) or whether they should be eating more fruits and vegetables (as recommended by MyPlate consumer messages [73]) even as they try to reduce overall daily calorie intake? The concept was that if results of a high-fidelity, low-attrition study confirmed the hypothesis that increased consumption of fiber-rich plant foods facilitated weight loss measured at 12 months follow-up, this randomized, controlled trial would permit confident causal inferences about the weight control benefit of encouraging low-income Americans to eat more fruits, vegetables, legumes, whole grains and nuts.

#### Forming the study cohort

African American and Latino adults in the U.S. have the highest age-adjusted rates of obesity relative to other major ethnic groups [74]. The investigators therefore partnered with a local FQHC, whose adult patients were 76% Latino and 13% African American based on pre-study demographic information reported to the state of California. Eligibility criteria included: 1) having a body mass index of between 27 and 40, 2) ability to communicate either in English or Spanish, 3) age 18 years or older, 4) willingness to change diet and exercise patterns, 5) willingness to accept randomization to either intervention group, and 6) ability to give informed consent. Exclusion criteria included: 1) pregnancy or intention to become pregnant in the next year, 2) having a major cardiac event or stroke-related medical procedure in last 6 months, 3) prior or planned bariatric surgery, 4) use of prescription medication for weight loss in the last 6 months, 5) chronic use of medications likely to cause weight gain or weight loss (e.g., antidepressants, mood stabilizers), 6) glucose control diabetes medications, 7) corticosteroids, 8) anti-seizure medications, 9) beta-blockers, 10) current cigarette smoking, 11) problem alcohol use, 12) psychiatric hospitalization in the last year, 13) plans to move from area in the next 12 months, 14) unstable angina, 15) blood pressure greater than 160/100 mm. The central rationale for these exclusions is that patients with these conditions would have difficulty adhering to intervention recommendations [75, 76]. Patients with uncomplicated type 2 diabetes could participate in the trial but only after being permitted to do so by their primary care provider. This last proviso was included at the behest of physicians who argued that patients newly diagnosed with diabetes could benefit from participation in a behavioral weight loss program and should not be barred from participation if they had not yet experienced complications associated with their disease.

#### Statistical power

To achieve satisfactory statistical power to detect the expected experimental difference in satiety, we relied on past literature involving use of a fruit and vegetable approach to facilitate weight loss [22]. With an effect size ((mean_baseline_ – mean_follow-up_)/mean standard deviation) of 0.52 ((53.5 mm – 46.7 mm)/13.2 = 0.52), the estimated per-condition sample size needed to detect an effect at 12 months follow-up was *n* = 72 [77]. To have the power necessary to evaluate differences in central body fat assessment at 12 months follow-up, we relied on three studies cited above [16, 17, 22], which yielded per-condition sample size estimates of *n* = 103 to *n* = 135. For the proposed two arm study and allowing for 20% attrition at 12 months, the prudent sample size target was set at *N* = 300.

### Patient recruitment

All participants were recruited in the FQHC’s waiting rooms. Accrual began on June 29, 2015 and ended February 29, 2016. Male and female research assistants, mostly college students, were all Spanish bilingual and were trained to select patients at random, regardless of perceived corpulence, despite knowing that patients would be ineligible to participate in the trial if their body mass index were less than 27. To minimize impact on clinic operations and patient flow, the research assistants were trained to subordinate the goal of rapid accrual to the goal of facilitating patient flow. If a patient was in the midst of completing the screening instrument when she/he was called up for their medical appointment, the research assistants were told to interrupt the screening and hope that the patient would return after the clinic visit to complete the screening. Average screening time was 7–8 min. If eligible patients agreed to enroll, they were randomly allocated to the MyPlate or Calorie Counting condition by a computer program in REDCap [78] only after providing written informed consent to participation, completing all baseline assessment questionnaires (research assistant delivered interviews), and providing anthropometric and blood pressure data. The baseline questionnaire was typically completed during that first encounter but, if necessary, was completed at a specially scheduled return visit. The patient’s primary care provider determined whether or not there were medical contraindications to the patient participating in the trial and otherwise restricted him/herself to encouraging the patient to enroll in the trial to address their overweight/obesity status. Randomization occurred after baseline assessment and enrollment activities to keep research staff blind to the patient’s experimental condition. Consent forms and questionnaires were written in English and Spanish and participants were given the option of receiving materials in the language of their preference. In addition, study participants were given the option of completing the Block Food Frequency Questionnaire at the same visit or by phone at a time of their convenience.

### Ethics approval and consent to participate

Study protocols were approved by the University of California, Los Angeles Institutional Review Board, IRB#14–001360. All participants provided signed consent.

### Consent for publication

Not applicable because no data for an individual participant were reported.

#### Study setting

Although initial contact with the patient was in the clinic waiting room, most of the health education sessions occurred off site. Two of the sessions took place in the patient’s home because those sessions were focused on how to make the home environment more supportive of healthier lifestyle choices. One group education session took place in the grocery store environment because that session focused on how to make typical food shopping more supportive of healthy food choices. Most of the coaching sessions took place on the phone at times convenient to the study participant. Group cooking sessions took place in offices of the FQHC or at community sites close to the FQHC.

#### Partnering FQHC

The leadership of the partnering FQHC was strongly supportive of the research, facilitating the recruitment and training of FQHC primary care providers. No patients could be enrolled in the trial without first obtaining the permission of their FQHC primary care provider. All primary care providers participated in a 1-h orientation to the study prior to participant recruitment. A list of exclusionary criteria was made available to the primary care provider for each study candidate to facilitate the provider’s decision whether or not there was information in the patient’s medical record contraindicating their participation in the weight loss trial.

#### Interventions

#### Choice of comparators

It has been established that overweight patients are highly interested in receiving advice from their primary care physicians about effective lifestyle change approaches to losing excess weight [79]. Below are descriptions of the two behavior change approaches used in this study.

#### Calorie counting approach

The traditional government recommendation to clinicians about effective advice to give to patients wanting to lose excess weight is well-reflected by the information at: https://www.niddk.nih.gov/health-information/weight-management/myths-nutrition-physical-activity http://win.niddk.nih.gov/publications/talking.htm#staff [80] or at: http://www.healthfinder.gov/prevention/ViewTopic.aspx?topicId=25 [81]. This information focuses on getting the overweight patient to deliberately adhere to an energy-deficit diet, where daily energy expenditure exceeds energy intake. The behavioral pathways to achieving a daily energy deficit include increased physical activity, careful monitoring of energy intake (i.e., calorie counting) and deliberate reduction of portions of foods commonly consumed to ensure achieving a daily calorie deficit. While there is some mention of substituting low-calorie foods such as fruits and vegetables for high-calorie foods, the focus is more on reducing the amount of current food choices rather than on changing the nature of the foods consumed [82]. With a couple of exceptions (see Table 1), the defining features of the Calorie Counting Approach were identical to the defining features of the diet prescribed in the Diabetes Prevention Program [15]. At the insistence of community members comprising our Community Advisory Board, the Calorie Counting approach was modified to include explicit encouragement to eat more fruits and vegetables regardless of calorie limits and to include explicit encouragement to limit consuming sugary beverages, even if within daily calorie limits. Community dietitians said that not to include these departures from the traditional calorie restriction approach would be professionally unacceptable, because they viewed failure to provide this information as withholding from the patient behavioral strategies now widely recognized as facilitating weight loss [83, 84]. The MyPlate rationale for recommending increased consumption of fruits and vegetables differed from the CC rationale in stressing how eating more fruits and vegetables promoted longer intervals between meals without hunger (satiety) but both approaches talked up the within-meal satiation benefits of eating more fruits and vegetables. Fruits and vegetables, among the major food groups, have the lowest energy density [85]. With respect to the specific daily calorie counting objective, participants in the CC condition were prescribed a daily calorie goal based on their body weight. Following the practice of the Diabetes Prevention Program, persons who weighed ≤114 kg (≤ 250 lbs) were prescribed 1200–1499 kcal/day and those > 114 kg (> 250 lbs) were prescribed 1500–1800 kcal/day. All participants were encouraged to aim for the lower end of their prescribed range of total daily calories consumed.

#### MyPlate approach

By contrast, the www.MyPlate.gov initiative [31] explicitly calls for changing the proportion of one’s plate that is devoted to different food groups, to eat more minimally processed fruits and vegetables relative to other food groups, to favor whole grains when grains are consumed, to replace high-fat dairy with low-fat or nonfat dairy, to replace sugary drinks with water, and to choose lower-sodium alternatives. Calorie counting was not encouraged and was not taught. The defining features of the MyPlate approach were the defining features of the Dietary Approach to Stop Hypertension (DASH) dietary pattern (See Table 1) [86]. This included encouragement to consume fewer snacks and sweets. The behavioral pathways to achieving a daily energy deficit using the MyPlate approach include: doubling typical intake of (minimally processed) fruits and vegetables, limiting intake of sugary beverages, engaging in moderately vigorous physical activity 150 min/week, and limiting sodium intake. The message that Americans can achieve a healthier weight by eating more of some foods is a relatively new message and one that would benefit from comparative assessment with the government’s more traditional calorie-counting, portion-control approach. Study attrition did not differ by experimental condition in a clinical trial of overweight adult women [22] and differential attrition was not expected to be a problem here. Protocols for both approaches have been well-detailed in recent clinical trials and have been associated with good study retention (78 to 86% retention) at 1 year follow-up [16, 17].

#### Additional intervention features common to both interventions

Both interventions recommended 150 min per week of moderate-to-vigorous physical activity. Participants in both conditions received upon enrollment a “gym-in-the-bag” that included a pedometer (Accusplit Digi-Walker Step Pedometer AE120XL)*,* self-monitoring forms for recording physical activity, a community resource guide, a digital weight scale for patients to monitor their weight at home (Smart Weigh Precision Digital bathroom scale), and stretch bands for resistance training (Black Mountain, from blackmountainproducts.com*).* The gym-in-the-bag of MyPlate participants but not CC participants also included a booklet of recipes illustrating tasty, creative ways to incorporate a broader range of fruits and vegetables into one’s regular food repertoire. Both interventions also featured the use of home environment audit instruments, designed to highlight physical cues (e.g. bowl of fresh fruit on the kitchen counter) and household routines (e.g., regular after-dinner neighborhood stroll) that have been previously associated with adherence to intervention recommendations [57, 87, 88].

#### Comparability of intervention exposure

Both conditions entailed the same number of contacts between the community health workers doing the lifestyle change coaching and the study participants regardless of assignment to condition. These contacts included two health education sessions in the home setting, two health education sessions in a group setting, and seven telephone coaching calls, all to be completed within 6 months of enrollment. Weekly debriefing calls between the investigators and the community health workers and the nesting of community health workers in each intervention ensured optimal adherence to the intervention protocols. Participants were assigned to a single community health worker, who interacted with the participant at all scheduled intervention sessions. A post-study evaluation included questions of study participants concerning the number of intervention sessions completed and their satisfaction with different components of the intervention.

#### Follow-up assessments

In this prospective one-year trial, follow-up assessments were conducted at 6-months and 12-months following the enrollment date and included measures similar to the baseline assessment, including the research assistant-administered interview and anthropometric measures. Food frequency questionnaires were administered only at baseline and 12 months follow-up.

### Study outcomes

#### Primary outcomes

The conventional primary outcome in previous trials of clinic-based weight loss interventions has been body weight [16, 17, 89, 90]. But successfully reduced body weight achieved at 12 months follow-up has not been enough to sustain a healthier body weight for 4 years or more [71] in part because the calorie restriction approach has been shown to be accompanied by increased hunger relative to a fruit and vegetable-supplemented approach [22]. For dietary changes to be sustained for a lifetime, not only does the excess weight need to be lost but the successful weight loss regimen also needs to leave the patient feeling satisfied after each day’s meals [91, 92]. Hunger and satiety ratings were patient-centered outcome measures identified as important by the patients and leaders in the focus groups and key informant surveys. Hence the investigators chose to include the everyday hunger scale used in previous research [22] as well as two additional questions about meal satisfaction and a feeling of fullness with meals [93] as primary endpoints. The choice of terms for assessing these facets of the satiety construct were evaluated using cognitive interviewing techniques to ensure that study participants correctly apprehended the meaning ascribed to these terms by the investigators. These terms were also vetted by focus group participants, the patient representatives and members of the Community Advisory Board.

The study [22] that the present study is patterned after included the following measure of hunger: “How hungry did you feel today?” The type of scale used was a 100 mm “Visual Analogue Scale (VAS).” The VAS consisted of a 100 mm line anchored at either extreme by “not at all hungry” and “extremely hungry.” Participants placed a hash mark on the line that best represented the level of hunger that they remembered having experienced the day before. Each VAS item was scored by measuring the distance from the left end of the line to the participant’s hash mark [94, 95]. In this trial, the hunger and two satiety-related VAS measures were prefaced by: “Take a moment to remember the last meal you ate yesterday.” Then, the wording of the hunger item was: “Thinking about yesterday, how hungry did you feel during the day?” The VAS scale was as described above. The wording of the meal satisfaction item was: “Thinking about the last meal you ate, how satisfied were you after you ate that meal?” The VAS scale was anchored by “Very satisfied” on the left and “Very unsatisfied” on the right. For analysis purposes, this scale was reverse-scored, so that high scores connoted satisfaction. Finally, the fullness question was: “Thinking about the last meal you ate, how full did you feel after you ate that meal?” The VAS scale was anchored by “Completely full” on the left and “Not at all full” on the right. For analysis purposes, this scale was reverse-scored, so that high scores connoted fullness. This variable was measured at baseline, 6- and 12-month follow-up assessments.

It was originally assumed that these three measures related to satiation/satiety would be sufficiently similar to justify including them in a single scale, to avoid the problem of inflation of type I error associated with multiple hypothesis-testing but the internal consistency (Cronbach’s alpha = 0.43) was unacceptably low. On the other hand, all three measures have been used in past nutrition research to represent the satiation/satiety constructs to good effect [22, 93, 96, 97], so all three were retained. To correct for the inflation of type I error in multiple comparisons [98], the investigators decided to use the Bonferroni correction, setting the nominal critical *p*-value to *p* = 0.0167 instead of *p* = 0.05.

While the hypothesis was that the MyPlate diet, with its doubling of fruits and vegetables, would yield greater satiation/satiety and reduced feeling of post-meal hunger than the DPP-like diet, a confounding contributor to feeling post-meal hunger is meal-skipping, both voluntary and involuntary. The lifestyle change coaches were trained to encourage breakfast eating in both conditions and to discourage meal skipping. For patients dependent on government food assistance, there may also be periods of involuntary hunger. Two questions about food insecurity were asked of all participants and used as covariates to help control for the hunger-generating effects of periodic meal skipping. Here food insecurity means a household-level economic and social condition of limited or uncertain access to adequate *food* [99]*. The specific questions were: “*In the last 12 months, did you ever eat less than you felt you should because there wasn’t enough money to buy food?” and “In the last 12 months, were you ever hungry but didn’t eat because you couldn’t afford enough food?” The answer options were “Yes,” “No,” “Refused,” or “Don’t know.”

Primary Patient Medical Outcome included two indicators of body fat composition: weight (kg) and waist circumference.

Anthropometric measures of body fatness are conventionally used to assess the impact of clinic-based weight loss interventions [16, 17]. Weight (kg) was measured at each assessment in the clinic setting. Weight in light indoor clothes without shoes was recorded by trained, certified staff using a high-quality digital scale (Tanita model BWB 800S). Participants were asked to remove any clothing accessories and heavy articles of clothing, (examples, watches, wallets, keys, cell phones, etc.). Weight was measured to the nearest 0.1 kg and duplicate measurements were made to ensure reliability. Scales were calibrated weekly using Troemner standardized weights (Troemner, Thorofare, NJ). The weight at screening/baseline was used to determine eligibility (27.0 < = BMI < = 40.4). The difference between body weight obtained at screening/baseline and 12 months follow-up was the primary patient medical outcome. Although easily measured by patients, body weight is an imperfect gauge of metabolically significant body fat because it can be influenced by exercise-induced hypertrophy of lean body tissue [100]. Waist circumference is arguably a better reflection of abdominal fat, which is a more consistent risk factor for metabolic disease than subcutaneous fat [101–104]. Participants’ waist circumference was therefore measured at each assessment. Waist Circumference (cm) was measured by trained staff using an anthropometric measuring tape (Gulick anthropometric tape) at a horizontal plane around the abdomen just above the uppermost lateral border of the right iliac crest (i.e. the top of the hip bone) [105]. Values were recorded to the nearest 0.1 cm and duplicate measurements were made to ensure reliability. Obesity cut points of 88 cm (women) and 102 cm (men) [105] were considered cut points separating those at significant risk of obesity-related disease from those at minimal risk. Ideally the waist circumference was measured against the skin but participants had the option of requesting that it be measured over clothing. If this option were taken, this option was recorded so that it could be used in analyses to correct for measurement error.

#### Prespecified secondary outcome measures

To replicate DASH trial blood pressure outcomes [18, 19] for the MyPlate condition, we included measurement of resting systolic blood pressure at each assessment for study participants in both experimental conditions. The study participant rested for 5 minutes before having the first blood pressure assessment using an automated sphygmomanometer that was calibrated regularly against a Life Source UA-767 Plus, A&D Medical digital blood pressure monitor. Blood pressure was obtained by trained data collectors according to a standard protocol, adapted from that used by the CDC [106]. Two measures were taken, 1 minute apart. If these two measures varied by more than 5 mm, then a third measure was taken and averaged with the preceding two in analyses.

#### Intervention check

Using the MyPlate icon (at www.choosemyplate.gov) the community health workers in the MyPlate intervention stressed the importance of filling half of one’s plate with (minimally processed) fruits and vegetables. CC participants were also encouraged to consume more fruits and vegetables but only because of their low energy density. All participants answered questions about how much of their average plate they filled with fruits and vegetables. The answer options were as follows: none, quarter plate, half plate, three quarters plate, and full plate.

#### Health-related quality of life and mental health

In theory, the high-satiety MyPlate approach of the MyPlate approach would lead over time to a lower sense of deprivation and hunger during active weight control efforts than traditional calorie restriction approaches and lead to enhanced health-related quality of life [40] and lower risk of depressiveness [107]. We therefore included the RAND-12 health-related quality of life scale [108, 109] and the Mental Health Index-5 (MHI-5) mental health scale [109]. The items of both scales were derived from the RAND-36 quality of life scale [110]. For the RAND-12 health-related quality of life measure, the convention is to take the 12 items, with answer options ranging from dichotomous items to six ordered options, and scale them such that the maximum score for each item is 100. High scores represent a high quality of life; low scores represent a low quality of life. Internal consistency: Cronbach’s alpha = 0.82.

Three of the MHI-5 items were shared with the RAND-12 and the remaining two items were taken from the RAND-36 [111]. The MHI-5 items included the following questions: ‘How much of the time during the last month have you: (i) been a very nervous person?; (ii) felt downhearted and blue?; (iii) felt calm and peaceful?; (iv) felt so down in the dumps that nothing could cheer you up?; and (v) been a happy person?’ All of these items included the following answer options: 1) all the time, 2) most of the time, 3) a good bit of the time, 4) some of the time, 5) a little of the time, 6) none of the time. Items were reverse-coded, as necessary, so that high scores represented greater mental health. As was done for the RAND-12 measures above, the answers were scaled so that the maximum possible mental health score for each measure was 100. Internal consistency: Cronbach’s alpha = 0.76.

#### Secondary outcome measures (not prespecified)

The scientific literature on behavior change weight loss programs has included a variety of behavioral, psychological and social measures as covariates. To optimize the comparability of our results with the results reported in the literature, we included the following covariates: 1) self-reported physical activity, 2) television watching as a proxy for sedentary behavior, 3) family support for healthy eating, 4) family support for leisure time physical activity, 5) food frequency questionnaire assessment of typical food choices in the last year, and 6) acculturation. Acculturation was included in part because the investigators were aware that many of the FQHC patients were immigrants and first generation immigrants tend to retain higher quality dietary patterns than U.S.-born co-ethnics [112–114]. The food frequency questionnaire was administered at baseline and 12 months follow-up. All the other covariates listed here were administered at baseline, 6 and 12 months follow-up. Inclusion of specific covariates in regression analyses was determined by theory, not by stepwise methods. Information about how these covariates were coded when included in regression analyses is provided below.

#### Physical activity (two indicators: 1) self-reported minutes of moderately vigorous-equivalent minutes of physical activity per week, 2) heart rate)

Advice to increase daily physical activity to at least 30 min of moderate to vigorous physical activity at least 5 days a week was given to participants in both conditions. Participants in both conditions received a “gym in the bag” that included a 10-min “Instant Recess™” DVD featuring fun dance routines, resistance bands, a pedometer, and charts with which to monitor progress. Self-report questions were taken from the International Physical Activity Questionnaire-short version [115] to assess the frequency and duration of different moderate and vigorous forms of physical activity. This 7-item questionnaire collects information on the time (i.e., number of days and average time per day) spent being physically active and measures vigorous-intensity activity, moderate-intensity activity, walking activity, and sitting in the last seven consecutive day period. An aggregate weekly number of moderately vigorous physical activity-equivalent minutes were calculated from these responses and entered into regressions as a continuous measure after truncation of outliers.

#### Objective measure reflective of physical fitness: heart rate

Heart rate has been used as a proxy measure of physical fitness that covaries reasonably well with peak oxygen uptake, the gold standard for fitness assessment [116]. The resting heart rate was obtained automatically during the blood pressure assessment, following a 5-min rest and expressed in beats per minute. When included in regression analyses, the resting heart rate was given as an integer between 40 and 110 beats per minute.

#### TV watching

Reducing time spent watching TV was particularly encouraged in the MyPlate condition because of evidence that fruit and vegetable intake was inversely associated with number of hours of TV watching per day [117]. This was a self-report item that asked, “Over the past 30 days, on average how many hours per day did you sit and watch TV or videos?” Answer options were: 0 h, < 1 h, 1 h, 2 h, 3 h, 4 h, 5 or more hours. When included in regression analyses, this measure was represented by dummy values ranging from 1 (0 h) to 7 (5+ hours), which were normally distributed.

#### Family social support for healthy eating

Study participants completed 8 items adapted from measures of family support for healthy eating [118], yielding a scale with acceptable reliability (Cronbach’s alpha = 0.81). The stem was: “During the last 3 months, my family (or members of my household)…” Various examples of supportive or unsupportive behaviors were then listed (e.g, “Encouraged me not to eat “unhealthy foods” (cake, salted chips) when I’m tempted to do so*,” and “*Commented if I went back to my old  eating habits”). Answer options were: 1) “None,” 2) “Rarely,” 3) “A few times,” 4) “Often,” and 5) “Very often.” As appropriate, items were reverse-scored so that high scores denoted high family social support for heathy eating. When included in regressions, this covariate was represented by its dummy values because its values were normally distributed.

#### Family social support for increased physical activity

Participants also completed 9 items adapted from measures of family support for daily physical activity [118], yielding a scale with acceptable reliability (Cronbach’s alpha = 0.81). The stem was: “During the past three months, my family (or members of my household)…” Various examples of supportive or unsupportive behaviors were then listed (e.g., “Exercise with me,” or “Complained about the time I spend exercising.”). Answer options were: 1) “None,” 2) “Rarely,” 3) “A few times,” 4) “Often,” and 5) “Very often.” As appropriate, items were reverse-scored so that high scores denoted high family social support for leisure-time physical activity. When included in regressions, this covariate was represented by its dummy values because its values were normally distributed.

#### Food and beverage choices

The Block Food Frequency Questionnaire (FFQ) [119] was administered to participants at baseline and 12-months follow-up but not at 6-months follow-up. Hence, analyses including the food and beverage consumption data from the FFQs necessarily ignored the 6-months anthropometric and survey data. However, specific questions about sugary beverage consumption on the survey questionnaire overlapped with questions asked on the FFQ. For these survey questions, it was possible to model changes in consumption of sugary beverages at both 6 months and 12 months. Most FFQ items were expressed in mean grams consumed per day, or per week, after taking into consideration the mean amount of the food consumed and the frequency with which it was consumed. In some cases, the vendor for the Block Food Frequency questionnaire created aggregate variables derived from aggregating the consumption data involving specific categories of foods, such as all sweet-tasting foods or all sugar-sweetened beverages. Finally, to control for variations in participants’ daily food consumption, the grams of total fruit- and vegetable-derived fiber were divided by the total grams of food consumed daily and multiplied by 1000 to yield a fruit and vegetable fiber index per kilogram of food consumed daily [120]. Variables derived from the Block FFQ that were not normally distributed were subjected to log transformation to make the resulting values more consistent with the assumption that all predictors were normally distributed when included as covariates in regression analyses.

### Dietary quality

Dietary quality was assessed using the DASH dietary quality score as previously operationalized, applied to the food frequency data [121]. In brief, the DASH score assigns extra points for high intake of fruit, vegetables, nuts and legumes, low-fat dairy products, and whole grains according to quintile rankings (i.e., participants in the lowest quintile are assigned 1 point and those in the highest quintile are assigned 5 points). For problematic food constituents typically consumed in excess, the point system was reversed. With regard to intake of sodium, sweetened beverages, and processed meats, participants with lower quintiles of intake scored higher points (i.e., the lowest quintile received a score of 5 and the highest a score of 1) [121]. The total possible score range was 8–40. Because baseline respondent burden was already high and the Block Brief Food Frequency Questionnaire measure required 20–30 min to complete, participants had the option of completing the Block FFQ assessment at home by phone with an interviewer after enrollment in the trial and after initial exposure to the intervention, which could have affected baseline food frequency responses, biasing subsequent change scores towards the null hypothesis.

### Acculturation as a moderator variable

Acculturation to U.S. cultural practices was assessed by seven psychometrically well-established language-focused questions [122, 123] such as: “I speak English at home” “I write in Spanish (e.g., letters, emails)” and “I watch Spanish language movies on television.” Answer options were: “Never, Rarely, Sometimes, Usually, Always.” These seven items had high internal consistency (Cronbach’s alpha = 0.94). These items were subjected to a principal components analysis with one factor summarizing the shared variance. This factor was then used to evaluate the impact of acculturation on study outcomes. For some analyses this acculturation factor was categorized into tertiles. The investigators included these items at baseline because of consistent literature indicating that acculturation to U.S. dietary practices was associated with less adherence to components of the MyPlate prescription (e.g., less daily fruit intake, less legume intake, greater consumption of sugar in food and beverages) [124] and because the investigators anticipated that many of the study participants would be immigrants. Measures of language preference, as used here, are useful proxies for assessing acculturation but not the only way for researchers to assess study participants’ acculturation level. A 2008 review of studies evaluating the association between acculturation and dietary practices found consistent inverse associations between diet quality and acculturation despite variations in how acculturation was measured [124].

#### Satisfaction with the weight control program

Process measures were included in the 6 months and 12 months follow-up assessments to gauge participant satisfaction with the weight loss intervention program that they had been randomly assigned to. There were two overall indicators of participants’ satisfaction with their weight control program. One consisted of the question, “How useful was the Healthy Weight Loss Program for helping you to lose weight?” Answer options were: “Very useful, Somewhat useful, Not useful.” The other consisted of the question, “Would you recommend this Program to your family members or friends?” Answer options were: “Definitely,” “Maybe,” and “No.” This latter question is considered to be effective in predicting the success of a new commercial product or service [125].

### Data analysis plan for this baseline paper

The planned analyses included a qualitative assessment of focus group discussions, a comparison of baseline characteristics between CC and MyPlate conditions, multivariate assessment of the association between dietary quality and satiation/satiety, and multivariate assessment of the association between dietary quality and acculturation.

## Results

### Qualitative data

Results from the focus group discussions and key stakeholder interviews suggested that the original design of the MyPlate community health worker-based home environment-focused lifestyle change intervention was sound but needed minor modifications. Participants in the Spanish-speaking focus group were particularly receptive to the idea of a community health worker coming into the home to advise residents about visual cues, home equipment and family routines that could, if adopted, increase residents’ adherence to federal nutrition and physical activity recommendations. While participants in the English-speaking focus group were more ambivalent about a community health worker coming into the home, when it came to specific behavioral strategies they were equally supportive. All key stakeholder interviewees supported the idea of having a community health worker making home visits to advise residents on ways to increase their adherence to recommended guidelines, some of them enthusiastically so. The overall impression from all of these data, then, was support for the general concept of the original MyPlate intervention design but specific cautions about employing such intervention strategies as: 1) expecting participants to start every day with a healthy breakfast, 2) expecting participants to serve only non-caloric beverages to guests, 3) encouraging household members to engage in gardening every week (if garden was accessible), and 4) expecting participants to sleep 7–8 h a night.

The MyPlate approach resonated with focus group members as a particularly appropriate vehicle for communicating nutritional priorities to the mostly low-income, mostly Latino TCC patient population because it required minimal literacy and numeracy skills. The CC approach, by contrast, requires the ability to read food labels (literacy) and the ability to track and add up daily calories consumed (numeracy). Moreover, the MyPlate approach emphasis on fruits, vegetables, legumes and nuts accorded well with immigrants raised on the traditional Mesoamerican diet of maize, beans (e.g., black, pinto, etc.) and squash (e.g., pumpkin, acorn squash, butternut squash) [126]. The use of bilingual, bicultural community health workers as the change agents rather than masters level health educators was also consistent with community health practices in low-income Latino communities [127, 128].

### Recruitment

**Fig. 2:**
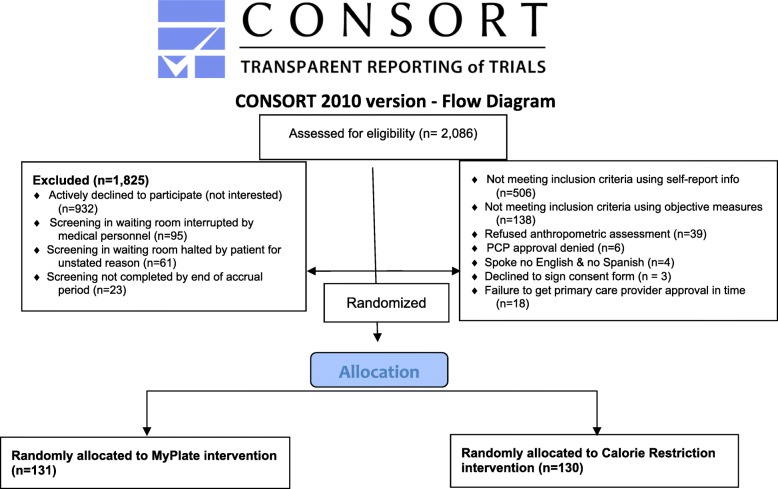
CONSORT flow diagram showing reasons for study ineligibility or withdrawal prior to baseline assessment

As indicated in the CONSORT diagram in Fig. 2, 2,086 patients were approached as they waited for their medical appointment in the clinic waiting room. Selection of patients for screening was random. Excluded from the study were 1,825 patients, mostly from patient’s lack of interest (51.1%), interruption during the screening to go in for their medical visit (9.8%), failing to meet inclusion criteria (35.3%) and miscellaneous reasons (3.8%), such as not speaking either English or Spanish. In total, 261 patients were enrolled and randomized to condition by computer-generated random assignment. Accrual ended short of the goal of enrolling 300 because the accrual process was taking longer than expected and because a sample size of 261 still provided adequate statistical power to test the main study hypotheses.

### Baseline characteristics

Table 2 includes participant baseline characteristics for the 261 participants who were fully enrolled in the trial. The table shows no statistically significant differences between experimental conditions on any demographic characteristics on the primary or secondary measures listed. The proportion of African American participants was only 8%, below the estimated 13% expected based on publicly available demographic statistics for the FQHC. The proportion of Latino participants was 86%, above the estimated 78% expected. Initial estimates were that one third of participants would be men but, as is commonly observed in community-based weight loss interventions [129], men were under-represented, with male participants comprising just 5 % of the study sample. Eighty-two percent of the sample was born outside of the U.S.; only 18% were born in the U.S.

**Table 2:** Descriptive characteristics of the baseline sample (*N* = 261)^a^

Measure	Total		MyPlate		Calorie Counting		*P*-value
	N	%	n	%	n	%	
Demographic measures							
Number of respondents	261	100%	131	50.2%	130	49.8%	
Sex							.54
Male	12	4.6%	5	3.8%	7	5.4%	
Female	249	95.4%	126	96.2%	123	94.6%	
Ethnicity							.71
Black or African American	20	7.7%	10	7.6%	10	7.7%	
Asian or Asian American	2	0.8%	1	0.8%	1	0.8%	
White/Caucasian	10	3.8%	7	5.3%	3	2.3%	
Hispanic/Latino	225	86.2%	112	85.5%	113	86.9%	
Native American	1	0.4%	0	0.0%	1	0.8%	
Other	3	1.2%	1	0.8%	2	1.5%	
Educational attainment							.43
Never attended / kindergarten only	6	2.3%	3	2.3%	3	2.3%	
Less than high school	120	46.0%	57	43.5%	63	48.5%	
High school / GED	76	29.1%	36	27.5%	40	30.8%	
Some college	53	20.3%	33	25.2%	20	15.4%	
College degree	5	1.9%	2	1.5%	3	2.3%	
Some grad sch/ post-college degree	1	0.4%	0	0.0%	1	0.8%	
Age (years); M = 41.8 (SD = 11.5)							.98
18–29 years	43	16.5%	23	17.6%	20	15.4%	
30–39 years	65	24.9%	32	24.4%	33	25.4%	
40–49 years	90	34.5%	44	33.6%	46	35.4%	
50–59 years	44	16.9%	23	17.6%	21	16.2%	
60+ years	19	7.3%	9	6.9%	10	7.7%	
Marital status							.95
Living without a partner/ single	125	47.9%	63	48.1%	62	47.7%	
Living as married/ married living together	136	52.1%	68	51.9%	68	52.3%	
Preferred language							.92
English	67	25.7%	34	26.0%	33	25.4%	
Spanish	194	74.3%	97	74.1%	97	74.6%	
Place of birth							.87
Born in US	47	18.1%	24	18.5%	23	17.7%	
Born outside US	213	81.9%	106	81.5%	107	82.3%	
Key outcome measures							
BMI (kg/m^2^); M = 33.3 (SD = 3.6)							.38
27 to < 30	54	20.7%	32	24.4%	22	16.9%	
30 to < 35	121	46.4%	60	45.8%	61	46.9%	
35 to < 40	73	28.0%	32	24.4%	41	31.5%	
40+	13	5.0%	7	5.3%	6	4.6%	
Waist circumference (cm); M = 102 (SD = 9.4)							.09
80 to < 90	19	7.3%	13	10.0%	6	4.6%	
90 to < 100	98	37.7%	52	40.0%	46	35.4%	
100 to < 110	89	34.2%	45	34.6%	44	33.9%	
110+	54	20.8%	20	15.4%	34	26.2%	
How much hunger did you feel yesterday? (mm)^b^; M = 49.1 (SD = 27.4)							.81
0 to < 25	39	14.9%	22	16.8%	17	13.1%	
25 to < 50	39	14.9%	20	15.3%	19	14.6%	
50 to < 75	131	50.2%	65	49.6%	66	50.8%	
75 to 100	52	19.9%	24	18.3%	28	21.5%	
Meal satisfaction did you feel yesterday? (mm)^b^; M = 32.1 (SD = 32.9)							.81
0 to < 25	124	47.5%	59	45.0%	65	50.0%	
25 to < 50	39	14.9%	20	15.3%	19	14.6%	
50 to < 75	54	20.7%	30	22.9%	24	18.5%	
75 to 100	44	16.9%	22	16.8%	22	16.9%	
How full did you feel after last meal yesterday (mm)^b^? M = 68.0 (SD = 28.9)							.52
0 to < 25	106	40.6%	49	37.4%	57	43.9%	
25 to < 50	55	21.1%	26	19.9%	29	22.3%	
50 to < 75	67	25.7%	38	29.0%	29	22.3%	
75 to 100	33	12.6%	18	13.7%	15	11.5%	
DASH dietary quality index							.008
Lowest tertile	75	32.6%	48	41.7%	27	23.5%	
Medium tertile	76	33.0%	36	31.3%	40	34.8%	
Highest tertile	79	34.4%	31	27.0%	48	41.7%	
Mental health index; M = 77.0 (SD = 18.6)							.48
0–64	69	26.5%	32	24.8%	37	28.2%	
65–80	70	26.9%	32	24.8%	38	29.0%	
81–92	70	26.9%	35	27.1%	35	26.7%	
93–100	51	19.6%	30	23.3%	21	16.0%	
Health-related Quality of Life; M = 74.2 (SD = 18.1)							.16
0–59	57	21.9%	27	20.9%	30	22.9%	
60–79	72	27.7%	42	32.6%	30	22.9%	
80–89	68	26.2%	27	20.9%	41	31.3%	
90–100	63	24.2%	33	25.6%	30	22.9%	
Key potential confounders							
Acculturation							.43
Lowest tertile	86	33.6%	39	30.5%	47	36.7%	
Medium tertile	85	33.2%	47	36.7%	38	29.7%	
Highest tertile	85	33.2%	42	32.8%	43	33.6%	
Food insecurity-not enough money to buy food							.18
No	203	78.1%	97	74.6%	106	81.5%	
Yes	57	21.9%	33	25.4%	24	18.5%	
Food insecurity-did not eat because could not afford to buy food							.49
No	225	86.2%	111	84.7%	114	87.7%	
Yes	36	13.8%	20	15.3%	16	12.3%	

^a^Sample size = 260 for Place of birth, waist circumference and 230 for DASH dietary quality because of missing data

^b^mm = millimeters on a 100 mm visual analogue scale

The intent of the MyPlate intervention was to increase patient dietary quality as measured by the DASH dietary quality score and thereby affect patient satiation/satiety. Despite the randomization of patients to experimental condition, there was a significant association of experimental assignment with the DASH dietary score, with higher scores observed in the MyPlate condition (chi square [2] = 9.75, *P* = .008) (See Table 2).

### Selected bivariate and multivariate associations of baseline characteristics with baseline primary and secondary outcome measures

Despite the restricted range of dietary quality expected at baseline, there could be significant cross-sectional associations between dietary quality and satiation/satiety. The baseline satiation/satiety measures were not associated with the patient’s DASH dietary score at baseline, however.

Similarly, there was no significant association between the baseline DASH dietary score and baseline body weight or waist circumference.

Implicit in the contrast between the MyPlate and CC approaches to weight loss is the expectation that change in dietary quality alone may facilitate weight loss in the MyPlate arm. However, nativity has been shown to be related to dietary quality, with U.S.-born participants typically reporting lower dietary quality [124, 130]. Because 82% of the study sample were foreign-born, the investigators evaluated dietary quality in relation to nativity. Forty-seven (18%) of participants were born in the U.S.; 185 (71%) were born in Mexico; 25 (10%) were born in the Central American countries of Honduras, El Salvador, Guatemala, and Nicaragua; two were from the Philippines; one was from Peru. Although there is overlap between the Peruvian diet and the Mesoamerican diet characteristic of Central America, the distinctive tubers, lupin beans, quinoa and distinctive tomato cultivars make the Peruvian diet sufficiently different from the diet of Mexico and Central America as to call into question classifying them in the same group. The one participant from Peru was therefore removed in diet-related analyses [131, 132]. The same reasoning applies to classification of the two participants from the Philippines in diet-related analyses [133].

To facilitate interpretation of results, analyses of the effects of nativity on dietary quality did not include the participants from the Philippines and Peru. With age, sex, ethnicity, educational attainment, marital status included as covariates, country of birth was significantly related to dietary quality, with Mexican-born participants reporting higher dietary quality than U.S.-born (DASH score_Mexican-born_ = 17.2, 95% CI: 16.6, 17.8 versus DASH score_U.S.-born_ = 14.98, 95% CI: 13.4, 16.6; *P* = .012). The DASH score for participants born in Central America was similar to that of Mexican-born participants and different from U.S.-born participants (DASH score_Central American-born_ = 17.6, 95% CI: 15.9, 19.4). This effect of nativity disappeared when acculturation was added as a covariate. This effect of nativity also disappeared in an analysis that did not include the 20 African American participants. As expected, the U.S.-born participants scored higher on U.S. acculturation than the foreign-born (Acculturation_foreign-born_ = − 0.26, 95% CI: 0.95, − 0.34 versus Acculturation_U.S.-born_ = 1.14, 95% CI: 0.93, 1.34; *P* < .0001). Baseline acculturation did not differ between experimental conditions.

Because fruit and vegetable fiber intake is critical to the satiety-enhancement benefit of the MyPlate approach, the two baseline conditions were compared on this outcome measure and were not significantly different (*P* > 0.10). Nor did the two conditions differ by daily water intake (*P* > 0.50). The two conditions did differ by daily sugary beverage intake, however, with participants in the MyPlate condition drinking just under one sugary beverage per day compared to nearly 1.3 beverages per day for participants assigned to the CC condition (sugary beverage frequency_MyPlate_ = 0.97, 95% CI: 0.79, 1.15 versus sugary beverage frequency_CC_ = 1.29, 95% CI: 1.06, 1.53, *P* < .05).

Food insecurity was clearly a factor in participants’ lives, with prevalence ranging from 13.8 to 21.95%. Baseline food insecurity did not differ, however, by experimental condition.

## Discussion

This study is the first to compare the calorie counting approach adapted from the DPP with the satiety enhancement-based approach of the DASH diet as summarized by MyPlate.gov. This study involves the participation of mostly low-income Latino and African American Primary Care Patients of a federally qualified health center. Community health workers from the community served as the intervention change agents. This paper described the study design, initial qualitative data, pilot data, and the enrolled sample’s baseline characteristics, study outcome measures, and baseline associations with key outcome measures at baseline.

### Key findings

#### Study limitations and strengths

Community dietitians objected to the investigators’ initial plans for the CC condition to make no recommendation about preferred food choices but instead to have the patient focus on limiting daily calorie intake, no matter what the source. They argued that the calorie restriction approach to weight control, which they advocated, had evolved since the design of the original Diabetes Prevention Program trial. A recent group-educated adaptation of the DPP, for example, includes a session that touts the weight loss benefits of eating more fruits and vegetables [134]. The CC condition was therefore modified and departed from the classical calorie restriction approach of the Diabetes Prevention Program but also more closely resembled the MyPlate intervention, thereby potentially reducing the effect size of the expected difference in satiation/satiety outcomes between the contrasting intervention approaches.

Two additional decisions were made in response to study participant requests to optimize their comfort level in participating in this study that increased measurement error. One was to permit the measurement of waist circumference over light clothing (e.g., a blouse, but not a jacket) instead of against the skin at the option of the participant. When the research assistant was a male, some female participants expressed a preference for the waist circumference to be measured over clothing. The second decision that increased measurement error was to permit participants the option to complete the Block Food Frequency Questionnaire at home (by phone interview) instead of the clinic, after all other baseline measures had been completed. Because participants completing the FFQ at home took more time to complete the FFQ than participants completing the FFQ at the clinic, some participants were exposed to some intervention sessions before completing the baseline Food Frequency Questionnaire, thereby possibly influencing their responses.

Another potential weakness was the observed association between acculturation / nativity and DASH dietary quality score, because U.S.-born participants’ daily food choices appeared to have more room for improvement than those of Mexican-born participants; the latter already adhere better to MyPlate-recommended food choices than do the U.S.-born participants. Ideally, future studies should take into consideration the lifestyle and eating habits of Latino immigrants before they move to the United States to help clarify the link between acculturation levels and dietary quality.

The strengths of the study included successful enrollment of a population considered “hard-to-reach,” namely a patient population composed primarily of low-income, Mexican immigrants. The four community health workers were full-fledged employees of the partnering FQHC, not the university, and two of them had several years of prior employment with the FQHC, facilitating the development of warm relationships between study participants and the community health workers. The multiple strategies for soliciting and incorporating community input into the design and vetting of intervention components and assessment instruments ensured that both interventions would be culturally acceptable to study participants.

## Conclusions

Two hundred and sixty-one low-income, overweight, mostly Spanish-speaking patients, were successfully enrolled and randomized to one of two experimental conditions in this comparative effectiveness behavior change weight loss trial. Baseline comparisons on key outcome measures between the CC and MyPlate conditions yielded minimal differences except for the DASH dietary quality score and sugary beverage intake. Baseline DASH dietary quality score and sugary beverage intake will be included as covariates in analyses of key outcome measures to correct for these baseline differences.

Consistent with previous literature [112–114], dietary quality was lower among those born in the U.S. and lower in those who opted to communicate in English rather than in Spanish.

In part because study participants were selected for obesity treatment, no association was observed between the dietary quality of their food choices and their satiation/satiety scores or body composition status at baseline. In more diverse populations, the scientific literature indicates that dietary quality is positively related to satiety [97, 135–137].

## Data Availability

The dataset supporting the conclusions of this article is available upon reasonable request by contacting the senior author or by accessing the data via the UCLA Fielding School of Public Health FSPH Public Data portal at: https://publicdata.ph.ucla.edu/pages/

## Abbreviations

BMI: Body mass index
CAB: Community advisory board
CC: Calorie Counting
cm: centimeters
CVD: Cardiovascular disease
DASH: Dietary Approach to Stop Hypertension
DPP: Diabetes Prevention Program
F&V: Fruits and vegetables
FFQ: Food frequency questionnaire
FQHC: Federally qualified health center
kg: kilogram
lbs.: pounds
MHI-5: Mental Health Index-5
MyP: MyPlate
UCLA: University of California, Los Angeles
VAS: Visual analogue scale
YMCA: Young Men’s Christian Organization

## References

[1] Centers for Disease Control (CDC) (2018). Table 53. Selected health conditions and risk factors, by age: U.S., selected years 1988–1994 through 2015–16.2018.

[2] Hales CM, Carroll MD, Fryar CD, Ogden CL (2017). Prevalence of obesity among adults and youth: United States, 2015–2016.

[3] Hales CM, Fryar CD, Carroll MD, Freedman DS, Aoki Y, Ogden CL (2018). Differences in obesity prevalence by demographic characteristics and urbanization level among adults in the United States, 2013-2016. JAMA-J Am Med Assoc.

[4] Finkelstein EA, Trogdon JG, Brown DS, Allaire BT, Dellea PS, Kamal-Bahl SJ (2008). The lifetime medical cost burden of overweight and obesity: implications for obesity prevention. Obesity..

[5] Tindall AM, Petersen KS, Kris-Etherton PM (2018). Dietary patterns affect the gut microbiome-the link to risk of Cardiometabolic diseases. J Nutr.

[6] Bastien M, Poirier P, Lemieux I, Despres JP (2014). Overview of epidemiology and contribution of obesity to cardiovascular disease. Prog Cardiovasc Dis.

[7] Jensen MD, Ryan DH, Apovian CM, Ard JD, Comuzzie AG, Donato KA (2014). 2013 AHA/ACC/TOS guideline for the management of overweight and obesity in adults a report of the American College of Cardiology/American Heart Association task force on practice guidelines and the obesity society. J Am Coll Cardiol.

[8] American Diabetes Association (2012). Standards of medical Care in Diabetes-2012. Diabetes Care.

[9] Cleeman JI, Grundy SM, Becker D, Clark LT, Cooper RS, Denke MA (2001). Executive summary of the third report of the National Cholesterol Education Program (NCEP) expert panel on detection, evaluation, and treatment of high blood cholesterol in adults (adult treatment panel III). JAMA-J Am Med Assoc.

[10] Chobanian AV, Bakris GL, Black HR, Cushman WC, Green LA, Izzo JL (2003). The seventh report of the joint National Committee on prevention, detection, evaluation, and treatment of high blood pressure - the JNC 7 report. JAMA-J Am Med Assoc.

[11] Maskarinec G, Grandinetti A, Matsuura G, Sharma S, Mau M, Henderson BE (2009). Diabetes prevalence and body mass index differ by ethnicity: the multiethnic cohort. Ethn Dis.

[12] Nazare JA, Smith JD, Borel AL, Haffner SM, Balkau B, Ross R (2012). Ethnic influences on the relations between abdominal subcutaneous and visceral adiposity, liver fat, and cardiometabolic risk profile: the international study of prediction of intra-abdominal adiposity and its relationship with Cardiometabolic risk/intra-abdominal adiposity. Am J Clin Nutr.

[13] Spanakis EK, Golden SH (2013). Race/ethnic difference in diabetes and diabetic complications. Curr Diabetes Rep.

[14] Estruch R, Ros E, Salas-Salvado J, Covas MI, Corella D, Aros F (2018). Primary prevention of cardiovascular disease with a Mediterranean diet supplemented with extra-virgin olive oil or nuts. N Engl J Med.

[15] Knowler WC, Barrett-Connor E, Fowler SE, Hamman RF, Lachin JM, Walker EA (2002). Reduction in the incidence of type 2 diabetes with lifestyle intervention or metformin. N Engl J Med.

[16] Wadden TA, Volger S, Sarwer DB, Vetter ML, Tsai AG, Berkowitz RI (2011). A two-year randomized trial of obesity treatment in primary care practice. N Engl J Med.

[17] Appel LJ, Clark JM, Yeh HC, Wang NY, Coughlin JW, Daumit G (2011). Comparative effectiveness of Weight-loss interventions in clinical practice. N Engl J Med.

[18] Appel LJ, Champagne CM, Harsha DW, Cooper LS, Obarzanek E, Elmer PJ (2003). Effects of comprehensive lifestyle modification on blood pressure - control main results of the PREMIER clinical trial. JAMA-J Am Med Assoc.

[19] Sacks FM, Svetkey LP, Vollmer WM, Appel LJ, Bray GA, Harsha D (2001). Effects on blood pressure of reduced dietary sodium and the dietary approaches to stop hypertension (DASH) diet. N Engl J Med.

[20] Vogt TM, Appel LJ, Obarzanek E, Mooe TJ, Vollmer WM, Svetkey LP (1999). Dietary approaches to stop hypertension: rationale, design, and methods. J Am Diet Assoc.

[21] U.S. Department of Agriculture (USDA) (2016). Dietary guidelines for americans 2015–2020.

[22] Ello-Martin JA, Roe LS, Ledikwe JH, Beach AM, Rolls BJ (2007). Dietary energy density in the treatment of obesity: a year-long trial comparing 2 weight-loss diets. Am J Clin Nutr.

[23] Jolly K, Daley A, Adab P, Lewis A, Denley J, Beach J (2010). A randomised controlled trial to compare a range of commercial or primary care led weight reduction programmes with a minimal intervention control for weight loss in obesity: the lighten up trial. BMC Public Health.

[24] Sifferlin A. Every Change Weight Watchers Just Made: Explained. Time Health. 2017:2017 Available from: http://time.com/4139180/weight-watchers-new-program/.

[25] Rautiainen S, Wang L, Lee IM, Manson JE, Buring JE, Sesso HD (2015). Higher intake of fruit, but not vegetables or Fiber, at baseline is associated with lower risk of becoming overweight or obese in middle-aged and older women of Normal BMI at baseline. J Nutr.

[26] Whigham LD, Valentine AR, Johnson LK, Zhang Z, Atkinson RL, Tanumihardjo SA (2012). Increased vegetable and fruit consumption during weight loss effort correlates with increased weight and fat loss. Nutr Diabetes.

[27] Wing RR, Tate DF, Gorin AA, Raynor HA, Fava JL (2006). A self-regulation program for maintenance of weight loss. N Engl J Med.

[28] James BL, Loken E, Roe LS, Rolls BJ (2017). The Weight-related eating questionnaire offers a concise alternative to the three-factor eating questionnaire for measuring eating behaviors related to weight loss. Appetite.

[29] Monsivais P, Rehm CD, Drewnowski A (2013). The DASH diet and diet costs among ethnic and racial groups in the United States. JAMA Intern Med.

[30] National Heart Lung and Blood Institute (NHLBI) (2014). Following the DASH eating plan.

[31] U.S. Department of Agriculture (USDA) (2015). Dietary Guidelines 2010 - Selected messages for consumers 2011.

[32] Esposito K, Marfella R, Ciotola M, Di Palo C, Giugliano F, Giugliano G (2004). Effect of a Mediterranean-style diet on endothelial dysfunction and markers of vascular inflammation in the metabolic syndrome - a randomized trial. JAMA-J Am Med Assoc.

[33] Lanza E, Schatzkin A, Daston C, Corle D, Freedman L, Ballard-Barbash R (2001). Implementation of a 4-y, high-fiber, high-fruit-and-vegetable, low-fat dietary intervention: results of dietary changes in the polyp prevention trial. Am J Clin Nutr.

[34] Pierce JP, Natarajan L, Caan BJ, Parker BA, Greenberg ER, Flatt SW (2007). Influence of a diet very high in vegetables, fruit, and fiber and low in fat on prognosis following treatment for breast cancer - the Women's healthy eating and living (WHEL) randomized trial. JAMA-J Am Med Assoc.

[35] Wojcicki JM, Heyman MB (2012). Reducing childhood obesity by eliminating 100% fruit juice. Am J Public Health.

[36] Deehan EC, Walter J (2016). The Fiber gap and the disappearing gut microbiome: implications for human nutrition. Trends Endocrinol Metab.

[37] Tolhurst G, Heffron H, Lam YS, Parker HE, Habib AM, Diakogiannaki E (2012). Short-chain fatty acids stimulate glucagon-like Peptide-1 secretion via the G-protein-coupled receptor FFAR2. Diabetes.

[38] Ledikwe JH, Rolls BJ, Smiciklas-Wright H, Mitchell DC, Ard JD, Champagne C (2007). Reductions in dietary energy density are associated with weight loss in overweight and obese participants in the PREMIER trial. Am J Clin Nutr.

[39] Rothberg AE, McEwen LN, Kraftson AT, Neshewat GM, Fowler CE, Burant CF (2014). The impact of weight loss on health-related quality-of-life: implications for cost-effectiveness analyses. Qual Life Res.

[40] Klem ML, Wing RR, McGuire MT, Seagle HM, Hill JO (1997). A descriptive study of individuals successful at long-term maintenance of substantial weight loss. Am J Clin Nutr.

[41] Lasikiewicz N, Myrissa K, Hoyland A, Lawton CL (2014). Psychological benefits of weight loss following behavioural and/or dietary weight loss interventions. A systematic research review. Appetite.

[42] Hall KD, Kahan S (2018). Maintenance of lost weight and long-term management of obesity. Med Clin N Am.

[43] McGuire MT, Wing RR, Klem ML, Seagle HM, Hill JO (1998). Long-term maintenance of weight loss: do people who lose weight through various weight loss methods use different behaviors to maintain their weight?. Int J Obes.

[44] Bonaccio Marialaura, Di Castelnuovo Augusto, Bonanni Americo, Costanzo Simona, De Lucia Francesca, Pounis George, Zito Francesco, Donati Maria Benedetta, de Gaetano Giovanni, Iacoviello Licia (2013). Adherence to a Mediterranean diet is associated with a better health-related quality of life: a possible role of high dietary antioxidant content. BMJ Open.

[45] Bandura A (1977). Self-efficacy - toward a unifying theory of behavioral change. Psychol Rev.

[46] Bandura A (2004). Health promotion by social cognitive means. Health Educ Behav.

[47] Katigbak C, Van Devanter N, Islam N, Trinh-Shevrin C (2015). Partners in Health: a conceptual framework for the role of community Health Workers in Facilitating Patients’ adoption of healthy behaviors. Am J Public Health.

[48] Murayama H, Spencer MS, Sinco BR, Palmisano G, Kieffer EC (2017). Does racial/ethnic identity influence the effectiveness of a community Health worker intervention for African American and Latino adults with type 2 diabetes?. Health Educ Behav.

[49] Spencer MS, Rosland AM, Kieffer EC, Sinco BR, Valerio M, Palmisano G (2011). Effectiveness of a community Health worker intervention among African American and Latino adults with type 2 diabetes: a randomized controlled trial. Am J Public Health.

[50] Pinto AM, Fava JL, Hoffmann DA, Wing RR (2013). Combining behavioral Weight loss treatment and a commercial program: a randomized clinical trial. Obesity.

[51] Bennett GG, Warner ET, Glasgow RE, Askew S, Goldman J, Ritzwoller DP (2012). Obesity treatment for socioeconomically disadvantaged patients in primary care practice. Arch Intern Med.

[52] Dixon KJL, Shcherba S, Kipping RR (2012). Weight loss from three commercial providers of NHS primary care slimming on referral in North Somerset: service evaluation. J Public Health.

[53] Jolly K, Lewis A, Beach J, Denley J, Adab P, Deeks JJ (2011). Comparison of range of commercial or primary care led weight reduction programmes with minimal intervention control for weight loss in obesity: lighten up randomised controlled trial. Br Med J.

[54] Truby H, Baic S, Delooy A, Fox KR, Livingstone MBE, Logan CM (2006). Randomised controlled trial of four commercial weight loss programmes in the UK: initial findings from the BBC “diet trials”. Br Med J.

[55] Norman GJ, Kolodziejczyk JK, Adams MA, Patrick K, Marshall SJ (2013). Fruit and vegetable intake and eating behaviors mediate the effect of a randomized text-message based weight loss program. Prev Med.

[56] Wadden TA, Butryn ML, Wilson C (2007). Lifestyle modification for the management of obesity. Gastroenterology.

[57] Gorin AA, Raynor HA, Fava J, Maguire K, Robichaud E, Trautvetter J (2013). Randomized controlled trial of a comprehensive home environment-focused Weight-loss program for adults. Health Psychol.

[58] Campbell KJ, Crawford DA, Salmon J, Carver A, Garnett SP, Baur LA (2007). Associations between the home food environment and obesity-promoting eating behaviors in adolescence. Obesity..

[59] Gorin AA, Phelan S, Raynor H, Wing RR (2011). Home food and exercise environments of Normal-weight and overweight adults. Am J Health Behav.

[60] Anzman-Frasca S, Savage JS, Marini ME, Fisher JO, Birch LL (2012). Repeated exposure and associative conditioning promote preschool children's liking of vegetables. Appetite.

[61] Hausner H, Olsen A, Moller P (2012). Mere exposure and flavour-flavour learning increase 2-3 year-old children's acceptance of a novel vegetable. Appetite.

[62] de Wild VWT, de Graaf C, Jager G (2013). Effectiveness of flavour nutrient learning and mere exposure as mechanisms to increase toddler's intake and preference for green vegetables. Appetite.

[63] Caton SJ, Ahern SM, Remy E, Nicklaus S, Blundell P, Hetherington MM (2013). Repetition counts: repeated exposure increases intake of a novel vegetable in UK pre-school children compared to flavour-flavour and flavour-nutrient learning. Br J Nutr.

[64] U.S. Department of Health and Human Services (USDHHS) (2018). Physical activity guidelines for americans.

[65] Westerterp-Plantenga MS, Verwegen CRT, Ijedema MJW, Wijckmans NEG, Saris WHM (1997). Acute effects of exercise or sauna on appetite in obese and nonobese men. Physiol Behav.

[66] Codella R, Luzi L, Terruzzi I (2018). Exercise has the guts: how physical activity may positively modulate gut microbiota in chronic and immune-based diseases. Dig Liver Dis.

[67] Monteiro MP, Batterham RL (2017). The importance of the gastrointestinal tract in controlling food intake and regulating energy balance. Gastroenterology.

[68] U.S. Department of Health and Human Services (USDHHS) (2008). 2008 Physical activity guidelines for americans.

[69] Young DR, Coughlin J, Jerome GJ, Myers V, Chae SE, Brantley PJ (2010). Effects of the PREMIER interventions on Health-related quality of life. Ann Behav Med.

[70] Opie RS, O'Neil A, Itsiopoulos C, Jacka FN (2015). The impact of whole-of-diet interventions on depression and anxiety: a systematic review of randomised controlled trials. Public Health Nutr.

[71] Schwarzfuchs D, Golan R, Shai I (2012). Four-year follow-up after two-year dietary interventions. N Engl J Med.

[72] Krueger RA, Casey MA (2009). Focus Groups - A Practical Guide for Applied Research - Fourth Edition Sage.

[73] U.S. Department of Agriculture (USDA) (2011). Dietary guidelines for americans - selected consumer messages.

[74] Ogden CL, Carroll MD, Kit BK, Flegal KM (2014). Prevalence of childhood and adult obesity in the United States, 2011-2012. JAMA-J Am Med Assoc.

[75] Grenard JL, Munjas BA, Adams JL, Suttorp M, Maglione M, McGlynn EA (2011). Depression and medication adherence in the treatment of chronic diseases in the United States: a meta-analysis. J Gen Intern Med.

[76] Shuter J, Bernstein SL (2008). Cigarette smoking is an independent predictor of nonadherence in HIV-infected individuals receiving highly active antiretroviral therapy. Nicotine Tob Res.

[77] Cohen J (1992). Statistical power analysis. Curr Dir Psychol.

[78] Harris PA, Taylor R, Thielke R, Payne J, Gonzalez N, Conde JG (2009). Research electronic data capture (REDCap)-a metadata-driven methodology and workflow process for providing translational research informatics support. J Biomed Inform.

[79] Pool AC, Kraschnewski JL, Cover LA, Lehman EB, Stuckey HL, Hwang KO (2014). The impact of physician weight discussion on weight loss in US adults. Obes Res Clin Pract.

[80] National Institute of Diabetes Digestive and Kidney Disorders (NIDDK) (2017). Talking with patients about weight loss: Tips for primary care providers.

[81] U.S. Department of Health and Human Services (USDHHS) (2017). Watch Your Weight.

[82] Johnston BC, Kanters S, Bandayrel K, Wu P, Naji F, Siemieniuk RA (2014). Comparison of Weight loss among named diet programs in overweight and obese adults a meta-analysis. JAMA-J Am Med Assoc.

[83] Bertoia ML, Mukamal KJ, Cahill LE, Hou T, Ludwig DS, Mozaffarian D, et al. Changes in intake of fruits and vegetables and weight change in United States men and women followed for up to 24 years: analysis from three prospective cohort studies. PLoS Med. 2015;12:1–20. 10.1371/journal.pmed.1001878 PMC4578962 26394033

[84] Malik VS, Pan A, Willett WC, Hu FB (2013). Sugar-sweetened beverages and weight gain in children and adults: a systematic review and meta-analysis. Am J Clin Nutr.

[85] Rolls BJ (2009). The relationship between dietary energy density and energy intake. Physiol Behav.

[86] Appel LJ, Moore TJ, Obarzanek E, Vollmer WM, Svetkey LP, Sacks FM (1997). A clinical trial of the effects of dietary patterns on blood pressure. N Engl J Med.

[87] Kong A, Schiffer L, Antonic M, Braunschweig C, Odoms-Young A, Fitzgibbon M (2018). The relationship between home- and individual-level diet quality among African American and Hispanic/Latino households with young children. Int J Behav Nutr Phys Act.

[88] Fulkerson JA, Friend S, Horning M, Flattum C, Draxten M, Neumark-Sztainer D (2018). Family home food environment and nutrition-related parent and child personal and behavioral outcomes of the healthy home offerings via the mealtime environment (HOME) plus program: a randomized controlled trial. J Acad Nutr Diet.

[89] Barnard ND, Cohen J, Jenkins DJA, Turner-McGrievy G, Gloede L, Green A (2009). A low-fat vegan diet and a conventional diabetes diet in the treatment of type 2 diabetes: a randomized, controlled, 74-wk clinical trial. Am J Clin Nutr.

[90] Elfhag K, Rossner S (2005). Who succeeds in maintaining weight loss? A conceptual review of factors associated with weight loss maintenance and weight regain. Obes Rev.

[91] MacLean PS, Bergouignan A, Cornier MA, Jackman MR (2011). Biology’s response to dieting: the impetus for weight regain. Am J Physiol-Regul Integr Comp Physiol.

[92] Montesi L, El Ghoch M, Brodosi L, Calugi S, Marchesini G, Grave RD (2016). Long-term weight loss maintenance for obesity: a multidisciplinary approach. Diabetes Metab Syndr Obes.

[93] Shearrer GE, O'Reilly GA, Belcher BR, Daniels MJ, Goran MI, Spruijt-Metz D (2016). The impact of sugar sweetened beverage intake on hunger and satiety in minority adolescents. Appetite.

[94] Martin CK, Rosenbaum D, Han HM, Geiselman PJ, Wyatt HR, Hill JO (2011). Change in food cravings, food preferences, and appetite during a low-carbohydrate and low-fat diet. Obesity.

[95] Flint A, Raben A, Blundell JE, Astrup A (2000). Reproducibility, power and validity of visual analogue scares in assessment of appetite sensations in single test meal studies. Int J Obes.

[96] Karl JP, Meydani M, Barnett JB, Vanegas SM, Goldin B, Kane A (2017). Substituting whole grains for refined grains in a 6-wk randomized trial favorably affects energy-balance metrics in healthy men and postmenopausal women. Am J Clin Nutr.

[97] Arguin H, Tremblay A, Blundell JE, Despres JP, Richard D, Lamarche B (2017). Impact of a non-restrictive satiating diet on anthropometrics, satiety responsiveness and eating behaviour traits in obese men displaying a high or a low satiety phenotype. Br J Nutr.

[98] Armstrong RA (2014). When to use the Bonferroni correction. Ophthalmic Physiol Opt.

[99] U.S. Department of Agriculture (USDA) (2016). Definitions of food security 2016.

[100] Jabekk PT, Moe IA, Meen HD, Tomten SE, Hostmark AT (2010). Resistance training in overweight women on a ketogenic diet conserved lean body mass while reducing body fat. Nutr Metab.

[101] Hou XH, Lu JM, Weng JP, Ji LN, Shan ZY, Liu J (2013). Impact of waist circumference and body mass index on risk of Cardiometabolic disorder and cardiovascular disease in Chinese adults: a National Diabetes and metabolic disorders survey. PLoS One.

[102] Qiao Q, Nyamdorj R (2010). Is the association of type II diabetes with waist circumference or waist-to-hip ratio stronger than that with body mass index?. Eur J Clin Nutr.

[103] Janiszewski PM, Janssen I, Ross R (2007). Does waist circumference predict diabetes and cardiovascular disease beyond commonly evaluated cardiometabolic risk factors?. Diabetes Care.

[104] Bowman K, Atkins JL, Delgado J, Kos K, Kuchel GA, Ble A (2017). Central adiposity and the overweight risk paradox in aging: follow-up of 130,473 UK biobank participants. Am J Clin Nutr.

[105] U.S. Department of Agriculture (USDA). Managing Overweight and Obesity in Adults - Systematic evidence review from the Obesity Exerpt Panel, 2013. Rockville, MD: U.S. Department of Health and Human Services; 2013. https://www.nhlbi.nih.gov/sites/default/files/media/docs/obesity-evidence-review.pdf. Accessed 18 July 2019.

[106] Centers for Disease Control and Prevention (CDC) (2009). National Health and Nutrition Examination Survey (NHANES) Anthropometry Procedures Manual.

[107] Liu XQ, Yan Y, Li F, Zhang DF (2016). Fruit and vegetable consumption and the risk of depression: a meta-analysis. Nutrition.

[108] Ware JE, Kosinski M, Keller SD (1996). A 12-item short-form health survey - construction of scales and preliminary tests of reliability and validity. Med Care.

[109] Strand BH, Dalgard OS, Tambs K, Rognerud M (2003). Measuring the mental health status of the Norwegian population: a comparison of the instruments SCL-25, SCL-10, SCL-5 and MHI-5 (SF-36). Nord J Psychiatr.

[110] Hays RD, Morales LS (2001). The RAND-36 measure of health-related quality of life. Ann Med.

[111] Ware JE, Sherbourne CD (1992). The MOS 36-item short form health survey (SF-36). 1. Conceptual framework and item selection. Med Care.

[112] Reininger B, Lee M, Jennings R, Evans A, Vidoni M (2017). Healthy eating patterns associated with acculturation, sex and BMI among Mexican Americans. Public Health Nutr.

[113] Liu JH, Chu YH, Frongillo EA, Probst JC (2012). Generation and acculturation status are associated with dietary intake and body weight in Mexican American adolescents. J Nutr.

[114] Batis C, Hernandez-Barrera L, Barquera S, Rivera JA, Popkin BM (2011). Food acculturation drives dietary differences among Mexicans, Mexican Americans, and non-Hispanic whites. J Nutr.

[115] Craig CL, Marshall AL, Sjostrom M, Bauman AE, Booth ML, Ainsworth BE (2003). International physical activity questionnaire: 12-country reliability and validity. Med Sci Sports Exerc.

[116] Van Mechelen W, Kemper HCG, Twisk JWR, Van Lenthe FJ, Post GB, Armstrong N (1997). Longitudinal relationships between heart rate, maximal oxygen uptake, and activity. Children and exercise XIX: promoting health and wellbeing.

[117] Lipsky LM, Iannotti RJ (2012). Associations of television viewing with eating behaviors in the 2009 Health behaviour in school-aged children study. Arch Pediatr Adolesc Med.

[118] Sallis JF, Grossman RM, Pinski RB, Patterson TL, Nader PR (1987). The development of scales to measure social support for diet and exercise behaviors. Prev Med.

[119] Block G, Wakimoto P, Jensen C, Mandel S, Green RR (2006). Validation of a food frequency questionnaire for Hispanics. Prev Chronic Dis.

[120] Cavallo DN, Horino M, McCarthy WJ (2016). Adult intake of minimally processed fruits and vegetables: associations with Cardiometabolic disease risk factors. J Acad Nutr Diet.

[121] Fung TT, Chiuve SE, McCullough ML, Rexrode KM, Logroscino G, Hu FB (2008). Adherence to a DASH-style diet and risk of coronary heart disease and stroke in women. Arch Intern Med.

[122] Marin G, Sabogal F, Marin BV, Oterosabogal R, Perezstable EJ (1987). Development of a short acculturation scale for Hispanics. Hisp J Behav Sci.

[123] Marin G, Gamba RJ (1996). A new measurement of acculturation for Hispanics: the bidimensional acculturation scale for Hispanics (BAS). Hisp J Behav Sci.

[124] Ayala GX, Baquer B, Klinger S (2008). A systematic review of the relationship between acculturation and diet among Latinos in the United States: implications for future research. J Am Diet Assoc.

[125] Reichfeld FF (2003). The one number you need to grow. Harv Bus Rev.

[126] Zizumbo-Villarreal D, Flores-Silva A, Colunga-GarciaMarin P (2014). The food system during the formative period in west Mesoamerica(1). Econ Bot.

[127] Lujan J, Ostwald SK, Ortiz M (2007). Promotora diabetes intervention for Mexican Americans. Diabetes Educ.

[128] Balcazar H, Wise S, Rosenthal EL, Ochoa C, Rodriguez J, Hastings D (2012). An ecological model using Promotores de Salud to prevent cardiovascular disease on the US-Mexico border: the HEART project. Prev Chronic Dis.

[129] French SA, Jeffery RW, Wing RR (1994). Sex differences among participants in a weight control program. Addict Behav.

[130] Bostean G, Roberts CK, Crespi CM, Prelip M, Peters A, Belin TR (2013). Cardiovascular health: associations with race-ethnicity, nativity, and education in a diverse, population-based sample of Californians. Ann Epidemiol.

[131] Perrault-Archambault M, Coomes OT (2008). Distribution of agrobiodiversity in home gardens along the Corrientes River, Peruvian Amazon. Econ Bot.

[132] Calvo L, Esquibel CR (2015). Decolonize your diet: plant-based Mexican-American recipes for Health and healing.

[133] Alejandro RG, Tettoni LI (2005). Authentic recipes from the Philippines.

[134] Kramer K, Kriska A, Orchard T, Semler L, Venditti E (2017). Diabetes prevention program group lifestyle balance.

[135] Perry CP, Keane E, Layte R, Fitzgerald AP, Perry IJ, Harrington JM (2015). The use of a dietary quality score as a predictor of childhood overweight and obesity. BMC Public Health.

[136] Knaapila AJ, Sandell MA, Vaarno J, Hoppu U, Puolimatka T, Kaljonen A (2015). Food neophobia associates with lower dietary quality and higher BMI in Finnish adults. Public Health Nutr.

[137] Hall KD, Ayuketah A, Brychta R, Cai H, Cassimatis T, Chen KY (2019). Ultra-processed diets cause excess calorie intake and weight gain: An inpatient randomized controlled trial of Ad Libitum food intake. Cell Metab.

